# Human Mesenchymal Stem Cell Therapy Reverses Su5416/Hypoxia-Induced Pulmonary Arterial Hypertension in Mice

**DOI:** 10.3389/fphar.2018.01395

**Published:** 2018-12-06

**Authors:** Allan K. N. Alencar, Pedro M. Pimentel-Coelho, Guilherme C. Montes, Marina de M. C. da Silva, Luiza V. P. Mendes, Tadeu L. Montagnoli, Ananssa M. S. Silva, Juliana Ferreira Vasques, Paulo Henrique Rosado-de-Castro, Bianca Gutfilen, Valéria do M. N. Cunha, Aline G. M. Fraga, Patrícia M R e Silva, Marco Aurélio Martins, Tatiana Paula Teixeira Ferreira, Rosalia Mendes-Otero, Margarete M. Trachez, Roberto T. Sudo, Gisele Zapata-Sudo

**Affiliations:** ^1^Programa de Pesquisa em Desenvolvimento de Fármacos, Instituto de Ciências Biomédicas, Universidade Federal do Rio de Janeiro, Rio de Janeiro, Brazil; ^2^Instituto de Biofísica Carlos Chagas Filho, Universidade Federal do Rio de Janeiro, Rio de Janeiro, Brazil; ^3^Instituto de Ciências Biomédicas, Universidade Federal do Rio de Janeiro, Rio de Janeiro, Brazil; ^4^Faculdade de Medicina, Universidade Federal do Rio de Janeiro, Rio de Janeiro, Brazil; ^5^Faculdade de Farmácia, Universidade Federal do Rio de Janeiro, Centro de Ciências da Saúde, Rio de Janeiro, Brazil; ^6^Instituto Oswaldo Cruz/FIOCRUZ, Rio de Janeiro, Brazil

**Keywords:** pulmonary arterial hypertension, cell proliferation, inflammation, apoptosis, human mesenchymal stem cell

## Abstract

**Aims:** Pulmonary arterial hypertension (PAH) is a disease characterized by an increase in pulmonary vascular resistance and right ventricular (RV) failure. We aimed to determine the effects of human mesenchymal stem cell (hMSC) therapy in a SU5416/hypoxia (SuH) mice model of PAH.

**Methods and Results:** C57BL/6 mice (20–25 g) were exposure to 4 weeks of hypoxia combined vascular endothelial growth factor receptor antagonism (20 mg/kg SU5416; weekly *s.c.* injections; PAH mice). Control mice were housed in room air. Following 2 weeks of SuH exposure, we injected 5 × 10^5^ hMSCs cells suspended in 50 μL of vehicle (0.6 U/mL DNaseI in PBS) through intravenous injection in the caudal vein. PAH mice were treated only with vehicle. Ratio between pulmonary artery acceleration time and RV ejection time (PAAT/RVET), measure by echocardiography, was significantly reduced in the PAH mice, compared with controls, and therapy with hMSCs normalized this. Significant muscularization of the PA was observed in the PAH mice and hMSC reduced the number of fully muscularized vessels. RV free wall thickness was higher in PAH animals than in the controls, and a single injection of hMSCs reversed RV hypertrophy. Levels of markers of exacerbated apoptosis, tissue inflammation and damage, cell proliferation and oxidative stress were significantly greater in both lungs and RV tissues from PAH group, compared to controls. hMSC injection in PAH animals normalized the expression of these molecules which are involved with PAH and RV dysfunction development and the state of chronicity.

**Conclusion:** These results indicate that hMSCs therapy represents a novel strategy for the treatment of PAH in the future.

## Introduction

Pulmonary arterial hypertension (PAH) is characterized by increased precapillary pressure as a consequence of exacerbated pulmonary artery (PA) wall remodeling and hypertrophy (Gnecchi et al., 2016). These are the main causes for the development of right ventricle (RV) dysfunction, subsequent irreversible RV failure, and sudden cardiac death in patients with PAH. PAH remains a “killer” because the current available medications that focus on lung vasodilation to reverse the increased PA pressure often fail to reverse the disease and the long-term survival of patients is poor (Lajoie et al., 2016), highlighting the urgent need for the identification of innovative therapeutic strategies.

Similar to cancer, PAH initially shows deregulated angiogenesis, high production and release of growth factors from vessel cells, strong resistance to apoptosis, alteration of cellular metabolism, and abnormal formation of an inflammatory environment within the vessel walls (Voelkel et al., 1998; Guignabert et al., 2013; Boucherat et al., 2017). In advanced stages of PAH, the obliteration of the vascular lumen due to excessive cell proliferation is accompanied by excessive vasoconstriction, thus chronically increasing the RV overload, a cardiac chamber that initiates one compensatory adaption. These pathophysiological events are followed by progressive RV enlargement, leading to heart failure. Accordingly, favorable actions in the RV are also required for better outcomes; therefore, there is a current search for pleiotropic interventional approaches with beneficial roles throughout the cardiopulmonary system (pulmonary vessel and cardiac cells).

As PAH progression occurs due to tissue damage of the lung vessels with a deleterious effect in the RV, we understand that regeneration of the cardiopulmonary system function and structure could be a suitable strategy to improve a patient’s prognosis and quality of life. In this regard, numerous basic studies have examined the benefit of different stem cell preparations, providing support for the clinical trials of cell therapy to regenerate lung tissue due to diseases in clinical practice (Walter et al., 2014; Ahn et al., 2015; Horie et al., 2016; Liu et al., 2016; McIntyre et al., 2016; Savukinas et al., 2016). However, there is a lack of preclinical studies showing the effects of stem cells in suitable rodent models of PAH.

Of all types of stem cells, mesenchymal stem cells are one of the few lineages that have been applied for immunomodulation and tissue repair due to their strong capacity for releasing paracrine factors that contribute to cell regeneration (Liang et al., 2014). The human placenta and umbilical cord blood are rich in mesenchymal stem cells (Erices et al., 2000; Bieback et al., 2004; Rus Ciuca et al., 2011), and it has been demonstrated that human mesenchymal stem cells (hMSCs) collected from these areas show a better efficacy against cell damage compared to mesenchymal stem cells isolated from other tissues and organs (Zhu et al., 2014, 2015).

The aim of the present study was to provide additional information regarding the use of stem cells for the treatment of lung diseases, particularly in the context of PAH and RV dysfunction, by administrating hMSCs from umbilical cords to mice with SU5416/hypoxia (SuH)-induced PAH, a current preclinical model that closely resembles human PAH (Tuder et al., 1994; Ciuclan et al., 2011; Vitali et al., 2014). SU5416 is a potent antagonist of the vascular endothelial growth factor receptor 2 (VEGFR2) (Sun et al., 1998) and stimulates endothelial cell apoptosis and hyperplastic phenotype selection after diverse stimuli, such as chronic hypoxia, ovalbumin challenge or elevated shear stress (Sakao and Tatsumi, 2011; Nicolls et al., 2012). In mice, administration of SU5416 associated to chronic normobaric hypoxia produces angioobliterative lesions characterized by smooth muscle proliferation, vascular rarefaction and perivascular fibrosis, which contribute to increase precapillary vascular resistance and RV afterload and hypertrophy (Ciuclan et al., 2011; Vitali et al., 2014).

## Materials and Methods

### Drugs and Reagents

SU5416 was purchased from Sigma-Aldrich (St. Louis, MO, United States). Antibodies directed against tumor necrosis factor-alpha (TNF-α), active caspase-3, and the receptor for advanced glycation end products (RAGE) were purchased from Abcam (Cambridge, MA, United States); p-38 mitogen-activated protein kinase (p-38 MAPK), phosphorylated p-38 mitogen-activated protein kinase (p-p-38 MAPK), extracellular-signal-regulated kinase 5 (ERK5), phosphorylated extracellular-signal-regulated kinase 5 (p-ERK5), and glyceraldehyde-3-phosphate dehydrogenase (GAPDH) were purchased from Cell Signaling Technology (Danvers, MA, United States). The fluorescent secondary antibodies were purchased from LI-COR (Lincoln, NE, United States). Mouse anti-alpha smooth muscle actin (α-SMA) was purchased from Sigma (St. Louis, MO, United States), and the secondary antibody goat anti-mouse IgG-Alexa546 was purchased from Molecular Probes (Eugene, OR, United States).

### Isolation of hSMCs

The use of umbilical tissue was approved by the Institutional Review Board. The umbilical tissue was obtained after the informed consent form was signed by the donor. The cord was cut into small pieces, and the blood vessels were removed and digested in collagenase solution. After washing, the cells were cultured in Dulbecco’s modified Eagle Medium:Nutrient Mixture F-12 (DMEM/F12) containing 10% fetal bovine serum and penicillin/streptomycin (10 U/mL; 100 μg/mL). Cells were used after at least three passages. On the day of the experiment, the vials containing cells were defrosted and washed three times in a solution of DNase I (0.6 U/mL, Ambion) in phosphate-buffered saline (PBS). Cells were resuspended in 50 μL of the same solution immediately before injection into the caudal vein.

### Immunophenotyping of Umbilical Cord Mesenchymal Stem Cells (UC-MSC)

UC-MSC at passage 3 were immunophenotyped by flow cytometry. Briefly, UC-MSC were harvested and incubated with the antibodies listed below (1:50 dilution) in PBS supplemented with 0.5% BSA for 20 min at 4°C in the dark. Afterward, the cells were washed with PBS 0.5% BSA and centrifuged at 300 × *g* for 5 min. The pellet cells were resuspended at 300 μL PBS and data acquisition was performed. The UC-MSC were stained with the following antibodies: lineage 2 FITC – CD3, CD14, CD19, CD56, CD20 (cat. 64397); CD34 FITC (cat. 348053); CD45 FITC (cat. 555482); CD73 APC (cat. 560847); CD90 PE-Cy5 (cat. 555597); CD105 PE (cat. 560839) and HLA-DR (cat. 551375). The respective isotypes were used as negative control. All antibodies were purchased from BD Pharmingen. Data acquisition was performed on a BD FACSAria II and the analyses were performed using FlowJo software version X (Supplementary Figure 1).

### Experimental Design

**FIGURE 1:**
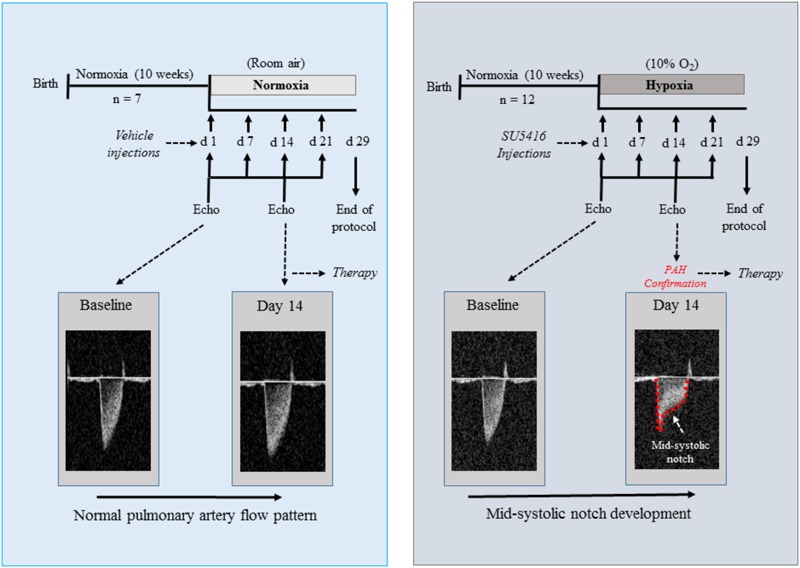
Schematic of the experimental design for the protocol of SuH-induced PAH. Echo Doppler images of the PA flow are showed for a normoxia and a SuH-induced PAH mice, with a mid-systolic notch being developed (red dashed line) in the PAH group at day 14 of protocol, as a sign of disease onset. SuH, SU5416/hypoxia; PA, pulmonary artery; PAH, pulmonary arterial hypertension.

All experiments were conducted in accordance with the Animal Care and Use Committee at the Universidade Federal do Rio de Janeiro. PAH was induced in mice by the SuH model. This model was chosen because it has been shown to be particularly useful when a stem cell- or progenitor cell-based therapy or pharmacotherapy is going to be evaluated (Vitali et al., 2014). Ten-week-old male C57BL/6J mice (20–25 g) were injected subcutaneously with SU5416 that was suspended according to validated procedures (Ciuclan et al., 2011; Vitali et al., 2014). The SuH mice were injected once weekly with SU5416 at 20 mg/kg body weight per dose and exposed to chronic normobaric hypoxia (10% O_2_; *n* = 12) in a ventilated acrylic chamber for 29 days in which nitrogen was injected (90%) under the control of an Oxycycler controller (BioSpherix, Lacona, NY, United States). Control mice were exposed to normoxia (room air at 21% O_2_; *n* = 7) and weekly vehicle injections (the same used to suspend SU5416). A baseline echocardiogram was performed before protocol initiation for all animal groups. Fourteen days after the start of SU5416 injections and chronic hypoxia, the mice underwent an echocardiographic evaluation for PAH confirmation. Doppler echocardiography was used to image the PA outflow waveform profile, and PAH establishment was confirmed by a change in the shape of the PA waveform, as described previously (Thibault et al., 2010; Gomez-Arroyo et al., 2012; Alencar et al., 2017). Figure 1 shows the experimental timeline used to characterize the evolution of PAH. Once disease onset had been confirmed, the SuH mice were divided into two groups: SuH + vehicle (0.6 U/mL DNaseI in PBS; SuH + Veh; *n* = 7) and SuH + hSMCs (*n* = 5). Vehicle (50 μL) or hMSCs (5 × 10^5^ cells suspended in 50 μL of vehicle) were injected into the caudal vein, and the animals were rapidly returned to the hypoxia chamber. The normoxia group similarly underwent echocardiography on the same day and was treated with a single injection of 50 μL of the same vehicle. Echocardiography was once again performed on day 28; and the protocol ended on day 29, when PAH was further assessed while the mice were under deep anesthesia [ketamine (80 mg/kg, i.p.) and xylazine (15 mg/kg, i.p.)] by hemodynamic measurements. Anesthesia depth was verified by pinching the animal’s paw with forceps. The thoracic cavity was opened, and a heparinized 21-G scalp weplast (Embramac) was inserted into the RV. The RV systolic pressure (RVSP) was measured with PowerLab monitoring equipment (AD Instruments, Sydney, NSW, Australia). Immediately after the hemodynamic measurements, the animals were sacrificed via exsanguination by cardiac puncture, and tissues were collected in order to perform hypertrophy, histology, and molecular pathway evaluations.

### Biodistribution of Cells

Fourteen days after SuH-induced PAH protocol, three mice received an intravenous injection of ^99m^technetium (^99m^Tc)-labeled hMSC (5 × 10^5^ cells) to detect the biodistribution of cells. Two hours after cell transplantation whole-body nuclear imaging was performed in a gamma camera (General Electric Medical Systems, Milwaukee, WI, United States) equipped with a high-resolution collimator. A 15% energy window centered on the 140 keV photo peak of ^99m^Tc was used. Additionally, after 24 h of ^99m^Tc-hMSC injection, mice were euthanized and the brain, heart, lungs, and liver were removed, weighed and the radioactivity was measured using a gamma counter (multi-crystal LB 2111 gamma counter, Berthold Technologies). The percentage of radioactivity analyzed per gram of organ (% radioactivity/g) was determined for each sample. After 2 h, whole-body nuclear imaging demonstrated radiotracer distribution mainly to the thorax and abdomen of the animals (lung and liver regions) (Supplementary Figure 2A) and minor retention at the site of injection. After 24 h of ^99m^Tc-labeled hMSC injection, radiotracer uptake was observed in the liver and in the cardiopulmonary system (Supplementary Figure 2B).

### Echocardiography

Experimental groups were anesthetized by a 2% isoflurane/oxygen mixture through a nose cone during spontaneous ventilation. Anesthetized spontaneously breathing animals were placed in a shallow left lateral decubitus position. The left hemithorax was shaved and prepared with acoustic coupling gel to increase probe contact. Room temperature was maintained at approximately 25°C to avoid hypothermia. Cardiac function was assessed by a high-resolution ultrasound imaging system equipped with a RMV-710B transducer with a frequency of 25 MHz and a fixed focal length of 15 mm mounted on an integrated rail system (Vevo 770, Visualsonics, Toronto, ON, Canada) as the procedure already published (Urboniene et al., 2010).

### Tissue Harvesting

The lungs were briefly washed in saline (before weighing), and the right lobes were immediately excised and frozen in liquid nitrogen for protein expression analysis. The left lobes were immersed in zinc formalin and processed for paraffin embedding, as published elsewhere (Alencar et al., 2017). The hearts were weighed, and the RV wall was separated from the left ventricle (LV) and septum (S). The ratio of the RV to the LV plus S weight (RV/LV + S) was calculated to determine the extent of RV hypertrophy.

### Membrane Preparations and Western Blot Analysis

Protein expression was measured as published elsewhere (Alencar et al., 2014). The whole western blot experiments are shown in the Supplementary Figure 3.

### Pulmonary Arteriole Muscularization

Five-μm paraffin sections of lungs were stained with orcein (1% in acid alcohol) for 30 min, washed in acid alcohol, and counterstained with picro-methyl blue (0.2% methyl blue in saturated picric acid solution) for 5 min before dehydrating and mounting with Entellan (Merck, Germany). In each lung, 15 arterioles of 30–100 μm in diameter were photographed with a digital camera (Canon A620, Canon, United States) coupled to an Axiostar optical microscope (Zeiss, Germany) under 1,000× magnification. Their mean wall thickness was expressed as the percentage of the vessel total cross-sectional area corresponding to the muscular wall (Alencar et al., 2017). Fully muscularized vessels were also counted under 400× magnification and normalized by the total section area.

Additional sections were immunostained for SMA expression by a modified mouse-on-mouse method (Goodpaster and Randolph-Habecker, 2014). Briefly, sections were rehydrated and blocked for 60 min in 5% bovine serum albumin in tris-buffered saline (TBS) and incubated for 2 h with antibody solution (1:500 anti-SMA, 1:1000 anti-mouse IgG-Alexa 546, and 1% bovine serum albumin in TBS previously mixed for 2 h). After extensive washing in TBS, the nuclei were stained with DAPI, and the slides were mounted with VectaShield (Vector Laboratories, Burlingame, CA, United States) and photographed under 200× magnification on a Zeiss Axiovert 200M fluorescence microscope (Zeiss, Oberkochen, Germany).

### Perivascular and Myocardial Fibrosis

Seven-μm paraffin sections of lungs were stained with picro-Sirius red, as described elsewhere (Alencar et al., 2017). For each lung, 10 arterioles of 30–100 μm in diameter were photographed with a digital camera (Canon A620, Canon, United States) coupled to an optical microscope (Axiostar, Zeiss, Germany) under 400× magnification. The perivascular collagen content was determined as the ratio of the perivascular collagen area to the vessel cross sectional area. Ten-μm frozen sections of hearts were immediately fixed in neutral buffered formalin and stained, similarly to the lung sections. RV interstitial fibrosis was assessed as the mean area of collagen in myocardial tissue under 1,000× magnification.

### ELISA Analysis

Murine cytokine levels were measured in heart samples by means of ELISA technique as previously described (Ferreira et al., 2013). Reagents from a commercial DuoSet kits R&D Systems (Minneapolis, MN, United States) were used according to the instructions of the manufacturer.

### Data Analysis

Data analysis was performed for all endpoints, and one-way analysis of variance was used to determine the significance of differences among groups. Significance of interactions between groups was determined by Tukey’s *post hoc* test. Pearson’s correlation was used to test for a relationship between the ratio of PA acceleration time to right ventricle ejection time (PAAT/RVET) and the RVSP. In addition, Western blot linear regressions analysis were performed. Differences for all tests were considered significant at *P* < 0.05. Analyses were performed using GraphPad Prism, version 6 (GraphPad, San Diego, CA, United States).

## Results

### Treatment With hMSCs Reduces the Functional and Histological Characteristics of SuH-Induced PAH

**FIGURE 2:**
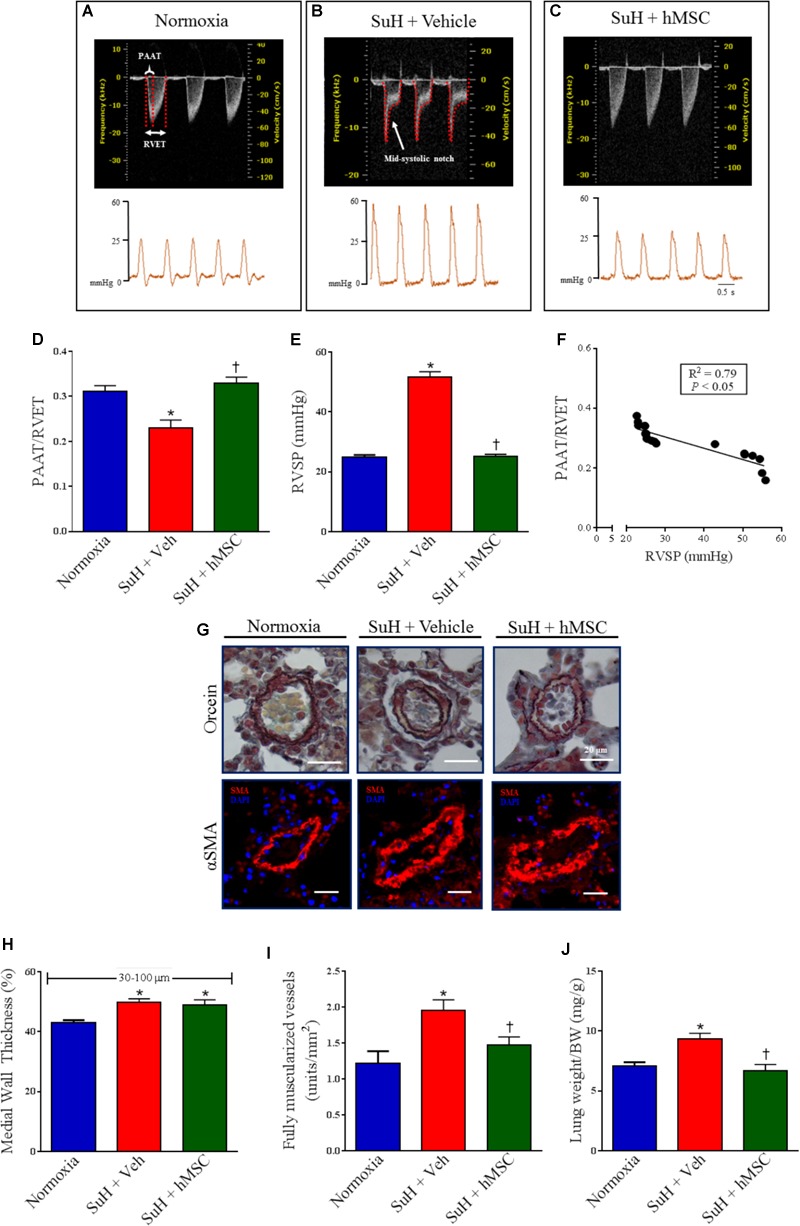
Effects of the hMSC therapy in the pulmonary vascular changes observed in SuH-PAH mice. **(A–C)** Representative images of PA outflow profile (upper) are presented for all groups 28 days after protocol initiation. Lower images are representative tracings of RVSP at day 29 of protocol. **(D)** Ratio between PAAT and RVET; **(E)** RVSP, and **(F)** linear regression between PAAT-to-RVET ratio and RVSP. **(G)** Representative orcein and immunostaining for α-SMA of the distal PAs exposed to normoxia or SuH protocol for 4 weeks. **(H)** Vessel wall thickness expressed as a percent of the total area of the vessel ranging between 30 and 100 μm in external diameter, **(I)** total of fully muscularized vessels, and **(J)** lung weigh-to-body weight ratio. Data represent the mean ± SEM (*n* = 5–7 mice per group). ^∗^*P* < 0.05 compared with normoxia group; ^†^*P* < 0.05 compared with SuH group treated with vehicle. Ordinary one-way ANOVA with multiple comparisons. hMSC, human mesenchymal stem-cell; SuH, SU5416/hypoxia; PAH, pulmonary arterial hypertension; PA, pulmonary artery; RVSP, right ventricular systolic pressure; PAAT, pulmonary artery acceleration time; RVET, right ventricle ejection time; α-SMA, alpha smooth muscle actin.

At the end of the experimental protocol, Doppler echocardiography was used to image the PA outflow waveform profile (Figures 2A–C, upper images). PAH establishment was confirmed by severe formation of a mid-systolic notch after chronic exposure to hypoxia and weekly injections of SU5416 (Figure 2B). Figures 2A–C (lower images) shows representative tracings of the right intracardiac pressures. Doppler echocardiography of the SuH group under light anesthesia demonstrated a reduction in the PAAT/RVET ratio (Figure 2D), compared to the normoxia group, indicating that the SuH mice developed PAH (*P* < 0.05). Under deep anesthesia, we further confirmed that the SuH mice developed RV overload as depicted by the greater RVSPs, compared to the normoxia group (Figure 2E; *P* < 0.05). Additionally, a close correlation was noted between the PAAT/RVET ratio and the RVSP (Figure 2F; *P* < 0.05). Importantly, hMSC therapy in the SuH mice normalized the PA flow profile (Figure 2C), significantly increased the PAAT/RVET ratio (Figure 2D), reduced the RV post-load, and decreased the RVSP (Figure 2E), compared to vehicle treatment by the end of the protocol (*P* < 0.05). The morphology of the distal PAs in the SuH mice was clearly different compared to that of the normoxia group (Figure 2G; *P* < 0.05). The SuH mice at 4 weeks after model induction exhibited a significant difference in the medial wall thickness of the PAs (Figure 2H; *P* < 0.05). Compared with the normoxia group, the SuH mice exhibited significant and severe muscularization of the distal PAs (Figure 2I) and a greater lung-to-body weight ratio (Figure 2J; *P* < 0.05). hMSC injection did not restrain medial vessel hypertrophy, but it significantly reduced the density of fully muscularized vessels and the lung weight (Figures 2H–J; *P* < 0.05).

### Therapy With hMSCs Reduced Distal PA Fibrosis and Normalized the Expression of RAGE in Lungs From SuH-Induced PAH Mice

**FIGURE 3:**
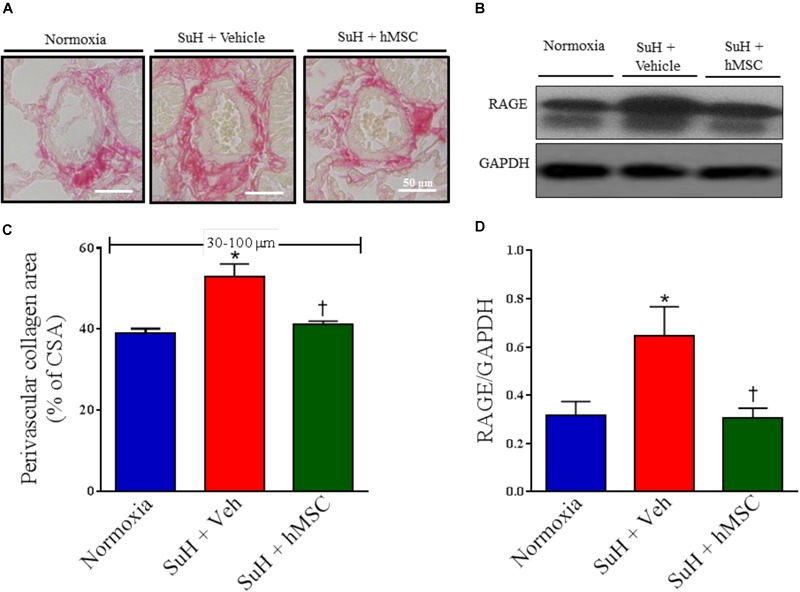
Effects of the hMSC therapy in the pulmonary vascular extracellular matrix deposition. **(A)** Picrosirius red staining. **(B)** Shows the Western blot analyses of RAGE in lungs from all animal groups. GAPDH was used for normalization. **(C)** Perivascular collagen area, and **(D)** quantification of RAGE expression. Each column and bar represent the mean ± SEM (*n* = 5–7 mice per group). ^∗^*P* < 0.05 compared with normoxia group; ^†^*P* < 0.05 compared with SuH group treated with vehicle. Ordinary one-way ANOVA with multiple comparisons. hMSC, human mesenchymal stem-cell; SuH, SU5416/hypoxia; RAGE, receptor for advanced glycation end products; CSA, cross section area.

Representative images of distal PAs showing the collagen content around the vessels as well as RAGE expression are presented in Figures 3A,B. Perivascular collagen deposition was significantly increased in the PAs from SuH mice, compared with the normoxia control group (Figure 3C; *P* < 0.05). Additionally, the RAGE protein levels were significantly greater in the lungs from the SuH-induced PAH mice, compared with the normoxia animals (Figure 3D; *P* < 0.05). After confirmation of PAH, treatment of the SuH group with hMSCs beneficially reversed perivascular fibrosis in the distal PAs, compared to the vehicle-treated SuH mice (Figure 3C; *P* < 0.05). Interestingly, the expression of RAGE, a receptor involved in the pathophysiological mechanisms of PAH, and extracellular matrix deposition in the PAs from SuH-induced PAH mice (Jia et al., 2017) were significantly increased in the lungs from PAH mice (*P* < 0.05). Moreover, RAGE expression was significantly normalized in the SuH mice that received hMSC therapy, compared to the vehicle-treated PAH mice (Figure 3D; *P* < 0.05).

### Therapy With hMSCs Normalized the Expression of Apoptosis, Inflammation, and Cellular Proliferation Markers in Lungs From Mice With SuH-Induced PAH

**FIGURE 4:**
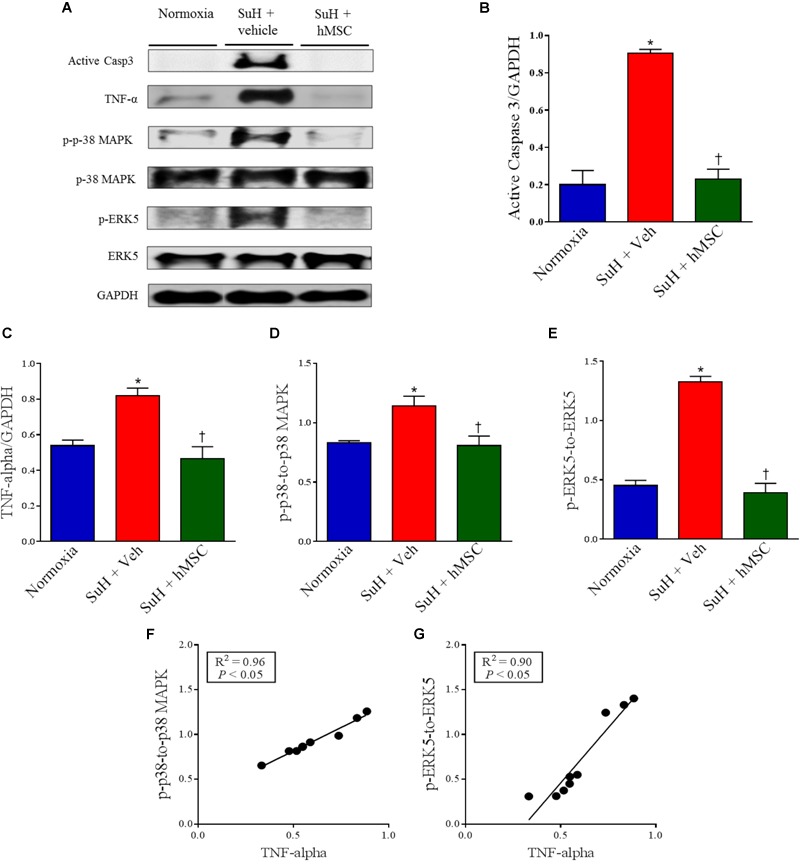
Effects of SuH model on the lung protein expression over 29 days of protocol and intravenous treatment with vehicle or hMSC at day 14 of protocol. **(A)** Shows the Western blot analyses of active caspase 3, TNF-α, p-p38 MAPK, p-38 MAPK, p-ERK5 and ERK 5 in lungs from normoxia or SuH mice, respectively. GAPDH was used for normalization. **(B)** Quantification of active caspase 3 expression. **(C)** Quantification of TNF-α. **(D)** Relative expression ratio of p-p-38 MAPK to p-38 MAPK and **(E)** relative expression of p-ERK5 to ERK5, respectively. **(F)** Linear regression between p-p-38 MAPK to p-38 MAPK ratio and TNF-α expression and **(G)** linear regression between p-ERK5 to ERK5 ratio and TNF-α expression. Each column and bar represent the mean ± SEM (*n* = 3–7 mice per group). *P* < 0.05 compared with normoxia group; ^†^*P* < 0.05 compared with SuH group treated with vehicle. Ordinary one-way ANOVA with multiple comparisons. hMSC, human mesenchymal stem-cell; SuH, SU5416/hypoxia; Active casp 3, active caspase-3; TNF-α, tumor necrosis factor alpha; p-p-38 MAPK, phosphorylated P-38 MAPK; p-38 MAPK, p-38 mitogen-activated protein kinase; p-ERK5, phosphorylated extracellular-signal-regulated kinase 5; ERK5, extracellular-signal-regulated kinase 5.

Western blot analysis of lung tissues from all experimental groups showed alterations of protein levels that are closely involved in the pathogenesis of PAH in the SuH mice, compared to the normoxia control group (Figure 4A; *P* < 0.05). Active caspase-3 and TNF-α levels were significantly increased in the vehicle-treated SuH mice, compared to their normoxia counterparts (Figures 4B,C; *P* < 0.05). The p-p-38 MAPK/p-38 MAPK and p-ERK5/ERK-5 ratios were also increased in the PAH group (Figures 4D,E; *P* < 0.05). Importantly, the TNF-α levels were significantly correlated with the intracellular kinases p-38 MAPK and ERK 5 (Figures 4F,G; *P* < 0.05). Fourteen days after the injection of hMSCs in the SuH mice with established PAH, we observed complete normalization of these PAH markers, compared to the vehicle-treated SuH group (Figures 4A–E; *P* < 0.05).

### Treatment With hMSCs Reduced Compensated RV Dysfunction and Right Chamber Concentric Hypertrophy in SuH Mice

**FIGURE 5:**
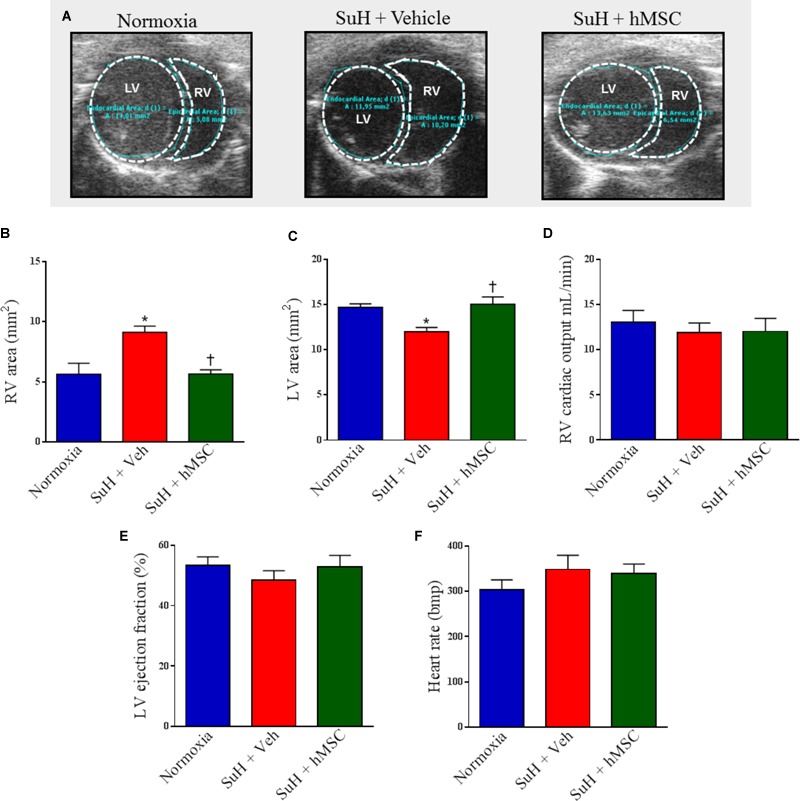
Effects of the treatment with vehicle or hMSC on heart structure and function of SuH-PAH mice. **(A)** Representative images of parasternal short-axis views obtained by B-mode echocardiography (all end-diastolic), **(B)** right ventricle area, **(C)** left ventricle area, and **(D)** right ventricular cardiac output 28 days after protocol initiation. **(E)** Left ventricle ejection fraction and **(F)** heart rate. Each column and bar represent the mean ± SEM (*n* = 5–7 mice per group). ^∗^*P* < 0.05 compared with normoxia group; ^†^*P* < 0.05 compared with SuH group treated with vehicle. Ordinary one-way ANOVA with multiple comparisons. hMSC, human mesenchymal stem-cell; SuH, SU5416/hypoxia; RV, right ventricle; LV, left ventricle.

**FIGURE 6:**
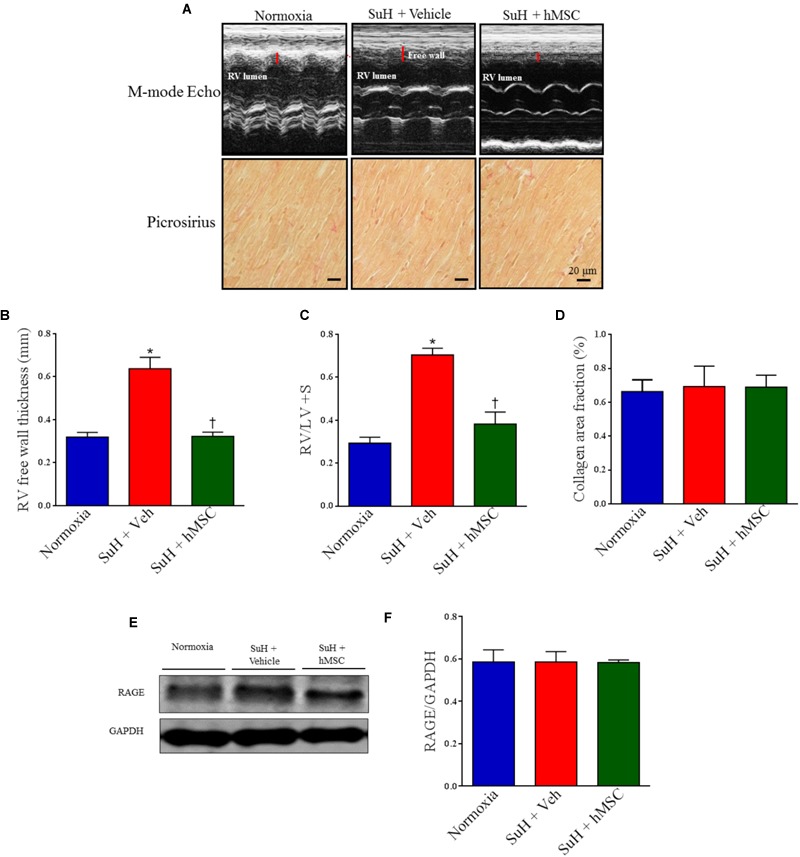
Effects of the therapy with vehicle or hMSC on RV free wall thickness, collagen volume fraction and RAGE expression in RV tissue from SuH-induced PAH or normoxia mice. (**A**, upper) Shows representative images of RV free wall obtained by M-mode echocardiography and (lower) picrosirius red staining under light microscopy (magnification 40×), showing collagen fibers in red in RVs from all animal groups. **(B)** RV free wall thickness, **(C)** RV-to-LV + septum ratio, **(D)** collagen volume fraction of RVs in relation to the tissue area. **(E)** Western blot analyses of RAGE in RV tissue from experimental groups. GAPDH was used for normalization. **(F)** Quantification of RAGE expression. Each column and bar represent the mean ± SEM (*n* = 3–7 mice per group). ^∗^*P* < 0.05 compared with normoxia group; ^†^*P* < 0.05 compared with SuH group treated with vehicle. Ordinary one-way ANOVA with multiple comparisons. hMSC, human mesenchymal stem-cell; SuH, SU5416/hypoxia; RAGE, receptor for advanced glycation end products; RV, right ventricle; LV, left ventricle; S, interventricular septum.

Figure 5A shows representative parasternal short-axis views obtained by B-mode echocardiography (all end-diastolic) for each group. The SuH model induced progressive RV remodeling and hypertrophy, as depicted by the increased RV area compared to that of the normoxia group (Figure 5B; *P* < 0.05), with a concomitant decrease in the LV area (Figure 5C; *P* < 0.05). As noted in Figures 5D,E, the mice did not develop severe heart failure, as the RV cardiac output and LV ejection fraction were not altered among animal groups by the end of the experimental protocol. However, an increased RV area resulted due to the long-term RV overload found in PAH, and, at day 28 of exposure to the SuH model, our mice had developed compensated (RV adaption) heart dysfunction. The heart rates of the animals were not changed at the end of the protocol (Figure 5F). Figure 6A shows the M-mode echocardiographic images and picrosirius red staining of RVs from all mouse groups. The higher degree of RV overload in the SuH-challenged mice led to marked RV concentric hypertrophy, as demonstrated by the significant increase in the RV free wall thickness in the SuH mice compared to the normoxia group (Figure 6B; *P* < 0.05). The ratio between the RV and LV + S weights also increased in the PAH group, compared to the normoxia group (Figure 6C; *P* < 0.05). Importantly, hMSC therapy reversed the deleterious effect of PAH-induced RV concentric hypertrophy in mice, as depicted by the lower RV free wall thickness and RV/LV + S ratio, compared to the vehicle-treated SuH mice (Figures 6B,C; *P* < 0.05). Interestingly, the RV collagen content was not altered among animal groups at this time point of the protocol (Figure 6D). Furthermore, Western blotting for RAGE expression in RV tissues from all groups showed no changes in this protein by the end of the protocol (Figures 6E,F).

### Therapy With hMSCs Normalized the Expression Levels of Apoptosis, Cellular Proliferation, and Inflammation Markers in Hearts From Mice With SuH-Induced RV Hypertrophy

**FIGURE 7:**
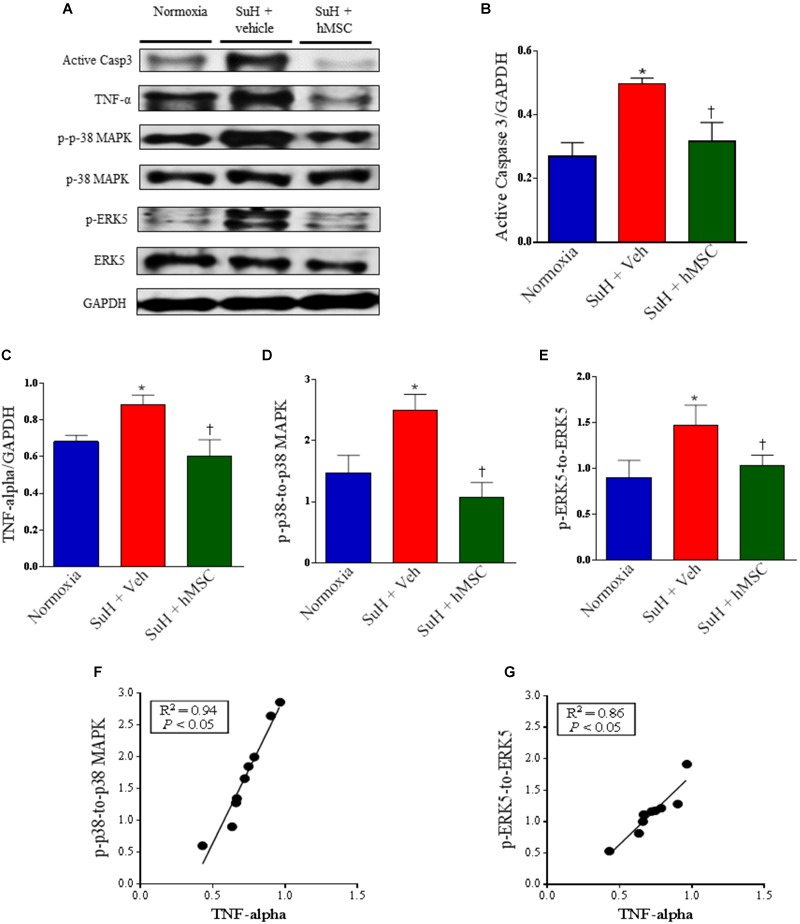
Effects of SuH model on the RV protein expression over 29 days of protocol and intravenous treatment with vehicle or hMSC at day 14 of protocol. **(A)** Shows the Western blot analyses of active caspase 3, TNF-α, p-p38 MAPK, p-38 MAPK, p-ERK5 and ERK 5 in RVs from normoxia or SuH mice, respectively. GAPDH was used for normalization. **(B)** Quantification of active caspase 3 expression. **(C)** Quantification of TNF-α. **(D)** Relative expression ratio of p-p-38 MAPK to p-38 MAPK and **(E)** relative expression of p-ERK5 to ERK5, respectively. **(F)** Linear regression between p-p-38 MAPK to p-38 MAPK ratio and TNF-α expression and **(G)** linear regression between p-ERK5 to ERK5 ratio and TNF-α expression. Each column and bar represent the mean ± SEM (*n* = 3–7 mice per group). ^∗^*P* < 0.05 compared with normoxia group; ^†^*P* < 0.05 compared with SuH group treated with vehicle. Ordinary one-way ANOVA with multiple comparisons. hMSC, human mesenchymal stem-cell; SuH, SU5416/hypoxia; Active casp 3, active caspase-3; TNF-α, tumor necrosis factor alpha; p-p-38 MAPK, phosphorylated P-38 MAPK; p-38 MAPK, p-38 mitogen-activated protein kinase; p-ERK5, phosphorylated extracellular-signal-regulated kinase 5; ERK5, extracellular-signal-regulated kinase 5.

Despite the fact that the SuH mice did not develop severe heart failure, we decided to evaluate the expression of markers for RV cell apoptosis (active caspase-3), exacerbated RV tissue injury and/or inflammation (TNF-α), and RV abnormal cell proliferation (p-38 MAPK and ERK5) (Figure 7A). The SuH-challenged mice showed an increase of active caspase-3 and TNF-α expression levels (Figures 7B,C; *P* < 0.05), with a concomitant increase in the p-p-38 MAPK/p-38 MAPK and p-ERK5/ERK-5 ratios (Figures 7D,E; *P* < 0.05), compared to their normoxia counterparts (*P* < 0.05). Similarly, to the lung tissue findings, in the RV myocardium, the TNF-α expression was also significantly correlated with the intracellular kinase levels of p-38 MAPK and ERK 5 (Figures 7F,G; *P* < 0.05). In addition to the reversion of RV wall hypertrophy showed in this work, the treatment of SuH mice with hMSCs normalized the expression patterns of all the molecules involved in the evolution of decompensated heart failure, compared to the vehicle-treated PAH animals (Figures 7A–F; *P* < 0.05).

**FIGURE 8:**
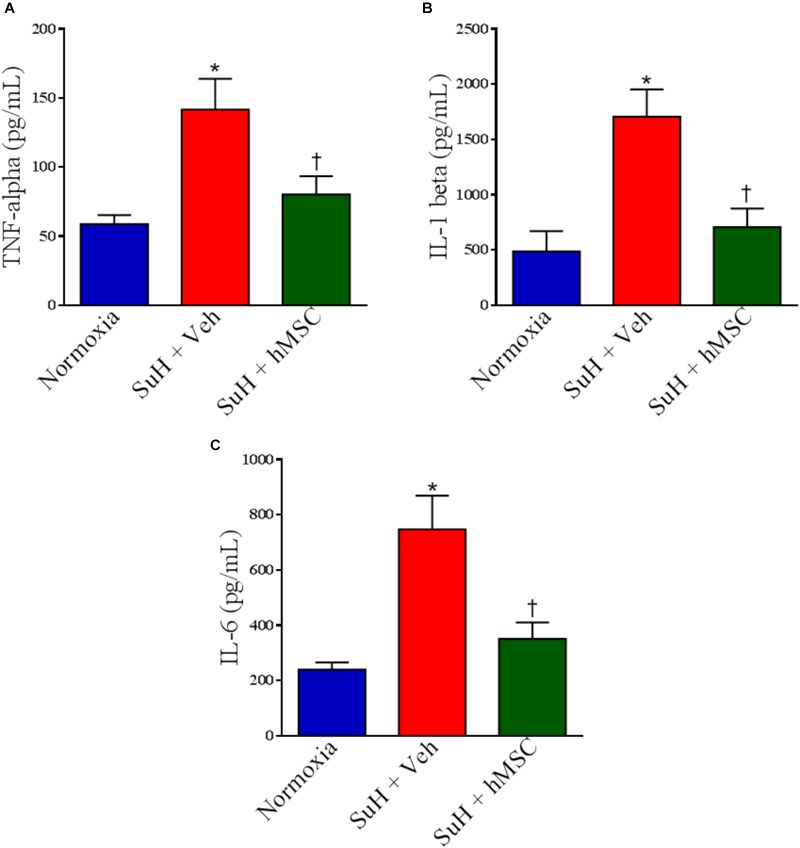
Effects of SuH model on the cytokine levels measured by ELISA assessment in RVs from experimental groups. **(A)** Levels of TNF-α, **(B)** levels of IL-1β, and **(C)** levels of IL-6. Each column and bar represent the mean ± SEM (*n* = 3–7 mice per group). ^∗^*P* < 0.05 compared with normoxia group; ^†^*P* < 0.05 compared with SuH group treated with vehicle. Ordinary one-way ANOVA with multiple comparisons. hMSC, human mesenchymal stem-cell; SuH, SU5416/hypoxia; TNF-α, tumor necrosis factor alpha; IL-1β, interleukin-1 beta, IL-6, interleukin-6.

Additionally, we have found significant differences in the levels of pro-inflammatory cytokines measured by ELISA assessment of RV homogenates obtained from all the animal groups. TNF-α, interleukin-1 beta (IL-1β), and interleukin-6 (IL-6) were all higher in RVs from PAH mice compared to control (*P* < 0.05), and hMSC injection importantly normalized the levels of these markers of cardiac inflammation (Figure 8; *P* < 0.05).

## Discussion

The main finding of this work was that hMSC therapy effectively ameliorated most of the alterations in the cardiopulmonary system of SuH-induced PAH mice. The evolution of PAH involves (1) microenvironmental changes in the distal PAs as the initial cause of the disease (pulmonary vessel remodeling and hypertrophy) with subsequent augmentation of pulmonary vascular resistance and (2) an insult to the RV myocardium that leads to RV failure (Boucherat et al., 2017). Therefore, we developed a schematic preclinical study in which we demonstrated the benefits of hMSC treatment on the echocardiographic, hemodynamic, histological, and molecular changes in the lungs and hearts from mice with PAH.

Our experimental protocol investigated the therapeutic activity of a stem cell lineage. First, we confirmed that mice in fact developed echocardiographic signs of PAH at 2 weeks after initiation of the SuH protocol. Subsequently, we injected hMSCs, which are now considered to be an important approach in the context of tissue regeneration (Liang et al., 2014; Gnecchi et al., 2016).

The multifactorial pathogenesis of PAH (Gnecchi et al., 2016) requires treatments with beneficial activity throughout the small circulation of patients diagnosed with this deleterious disease. Thus, acting on single targets might not be the best choice. In this work, we did not investigate the mechanisms by which hMSCs promote tissue repair, a topic that has already been well explored in the research field (Erices et al., 2000; Liang et al., 2014; Ahn et al., 2015; Gnecchi et al., 2016; Horie et al., 2016; Liu et al., 2016; McIntyre et al., 2016; Savukinas et al., 2016), but we specifically probed whether these cells could ameliorate the main changes that occur during PAH onset, including PA cell apoptosis, exacerbated vessel cell proliferation, remodeling, and inflammation. Additionally, we aimed to determine if this therapy could be considered as a pleiotropic approach—that is, if it might produce simultaneously more than one benefit in the cardiopulmonary system—through further repair of RV injury.

First, we confirmed that our SuH mice had developed PAH based on the echocardiographic and hemodynamic alterations observed during the evolution of the protocol. We combined the non-invasive technique of echocardiography, which can be used to estimate acute and chronic increases in the RVSP with a high sensitivity and specificity (Thibault et al., 2010), with the invasive measurement of the right chamber pressures. Anesthesia in mice might change the hemodynamic state of cardiac function (Roth et al., 2002), and it has been demonstrated that it is important to use light anesthesia rather than deep anesthesia for cardiac evaluation in mice (Thibault et al., 2010). As the PAAT/RVET ratio measured using light anesthesia correlated closely with the RVSP measured under deep anesthesia in our experiments, we assumed that PAH onset was suitably confirmed in both of our *in vivo* experiments.

The *in vivo* pulmonary circulation hemodynamic data changes could be explained by our histological findings showing that the distal PA walls had undergone remodeling and hypertrophy by the end of the experiments. Importantly, our Western blot data explained that the wall hypertrophy occurred as a consequence of the increased PA cell apoptosis induced by the SuH model, as depicted by the higher expression of active caspase-3 in lungs from SuH mice treated with vehicle. It has been shown that the endothelial apoptosis in PAH is mainly executed through activation of the caspase-3 axis, with subsequent cell death (White et al., 2014). Therefore, apoptosis could be an initiating mechanism for PAH by leading to vessel obliteration due to degeneration of endothelial cell structures (Zhao et al., 2005). Excessive loss of endothelial cells promotes the development of an apoptosis-resistant and hyperproliferative cell phenotype, which are features of later stages of PAH (Voelkel et al., 2002). As we measured active caspase-3 expression at 29 days after protocol initiation, we propose that its higher expression in the lungs from our PAH mice demonstrates that the PA cells were already resistant to apoptosis as the vessel wall thickness was increased, indicating deregulated vessel cell proliferation. Concomitantly, we observed higher phosphorylation of the intracellular mitogen-activated protein kinases p-38 MAPK and ERK5 in the lungs from the SuH animals, molecules that are involved in the cell proliferation process after an external stimulus (Nithianandarajah-Jones et al., 2012).

Endothelial cell-induced pulmonary vascular smooth muscle cell growth was greater in tissues from PAH patients than from controls (Eddahibi et al., 2006). Dysregulation of this process and excessive release of growth factors by endothelial cells are intrinsic abnormalities linked to PAH pathogenesis (Eddahibi et al., 2006). Indeed, abnormal communication between endothelial cells and other vascular cells in PAH may occur due to loss-of-function of the endothelium. PAH is among many pathophysiological conditions that are linked to inflammation (Schermuly et al., 2011), and the higher expression of TNF-α in the lungs from our PAH mice corroborates with this statement. TNF-α may also induce cell proliferation by activation of its tyrosine kinase receptor (Schermuly et al., 2011); thus, it is a molecule that is involved in the stimulation of intracellular kinases during the progression of PAH. Interestingly, the TNF-α levels were significantly correlated with p-38 MAPK and ERK5 protein expression in the lungs from our mice, indicating a role for TNF-α activation of PA cell proliferation during the lung vessel wall remodeling process.

Fibroblasts are cells that play important roles in PAH pathogenesis by responding to injury and chemoattraction via endothelium-derived growth factors. Rapid migration of fibroblasts to the injured vessel leads to formation of the neointimal layer by the release of collagen, and, most importantly, fibroblast transdifferentiation into other cell types, including myofibroblasts, an abnormal type of pulmonary vascular smooth muscle cells that contributes to muscularization of the distal vessels (Sartore et al., 2001; Sisbarro et al., 2005; Sakao et al., 2009). In our work, we showed that the SuH mice developed severe muscularization of the distal arterioles, with a concomitant increase in perivascular collagen deposition. A higher RAGE expression was also observed in our SuH mice. Of note, it has been described recently that this receptor is involved in the development of hypoxia-induced PH by increasing extracellular matrix deposition in pulmonary arteries from mice with PH (Jia et al., 2017). Our study showed, for the first time, that hMSC injection effectively reduced the muscularization of distal PAs, perivascular collagen content, and inflammation, with subsequent normalization of the intracellular phosphorylation of p-38 MAPK and ERK5 proteins. However, at this time of the protocol, the activity of hMSCs in the pulmonary tissue was not enough to normalize the distal PA wall thickness.

In our model of SuH-induced PAH, we observed that obliteration of the pulmonary vascular lumen resulted in RV adaption and hypertrophy, as depicted by the greater RV indices of hypertrophy measured by echocardiography and the tissue weight ratio. Despite the fact that we did not observe RV and global heart failure in our mice, based on our molecular findings at this time of the protocol, we assume that the RV cells were in a state of initial compensation that could be followed by progressive and irreversible RV enlargement. In a more prolonged experimental SuH exposure, RV failure would likely occur.

The myocardial collagen content and RAGE expression did not differ among our animal groups, but the RV hypertrophy and injury might be explained by the increased levels of TNF-α and the higher phosphorylation of the intracellular p-38 MAPK and ERK-5 proteins.

TNF-α is an important mediator of RV hypertrophy (Kubota et al., 1997; Smith et al., 2001; Jobe et al., 2009). Recently, the importance of TNF-α in the transition from compensated RV hypertrophy to decompensated RV failure has been described (Tang et al., 2015). Furthermore, MAPK pathways couple intrinsic and extrinsic signals to hypertrophic growth of cardiomyocytes (Nicol et al., 2001). The significant correlation between TNF-α and both p-38 MAPK and ERK5 in the RV tissue from our experimental groups corroborates with these findings, as the higher levels of p-38 MAPK and ERK5 phosphorylation may contribute to exacerbated cell proliferation, as commented earlier.

As the RV function is the main determinant of life expectancy in patients with PAH, we decided to further measure TNF-α and other cytokine levels, as IL-1β and IL-6, in hearts from our animal groups, all of these involved with cardiac inflammation and damage. Corroborating with the Western blot data for TNF-α, its levels were also greater in the SuH-induced PAH mice’s RVs than in normoxia group when measure by ELISA assay. Increased levels of IL-1β and IL-6 in RVs from SuH mice indicate the installation of a pro-inflammatory environment in the myocardium, and it has been described that these cytokines are involved with development of heart failure (Bujak and Frangogiannis, 2009; Heresi et al., 2014).

The greater expression of active caspase-3 in the RV tissue from SuH mice treated with vehicle additionally indicates that the apoptosis process had started at this time of the protocol, a finding that contributes to our statement that the RV from our PAH mice was transitioning to a failing phenotype.

It is also important to address that hMSCs can persist long-term in the RV by intracoronary injection and are a potential cell source for tissue repair in RV dysfunction induced in a large animal model of pulmonary hypertension (Badr Eslam et al., 2017). Herein, we expanded these beneficial findings on hMSCs for the whole cardiopulmonary system, now in a suitable rodent model of PAH. A strong paracrine capacity has been proposed as the principal mechanism that contributes to tissue repair promoted by mesenchymal stem cells (Gnecchi et al., 2016), and our innovative data show that mice with PAH indeed have their lungs and RV repaired by this cytotherapy.

The intravenous delivery of MSC has been proven to be safe in adult patients with different clinical conditions (Lalu et al., 2012) and, up to now, there is no report of tumor formation from infused MSC in preclinical *in vivo* studies and clinical trials (Barkholt et al., 2013). A few studies, however, have shown that human MSC can undergo spontaneous malignant transformation after long-term cultures and that the transformed cells can give rise to tumors in immunodeficient mice (Rosland et al., 2009; Pan et al., 2014), indicating that new approaches are needed for the assessment of the tumorigenic potential of MSC-based products (Yong et al., 2018). In our studies, we have used MSC cultured for no longer than five passages and we never detected any tumors in different animal models.

## Conclusion

In conclusion, our findings suggest that hMSCs normalize cell proliferation as well as attenuate tissue inflammation and injury in lungs and hearts from PAH mice, with a subsequent beneficial effect on the cardiopulmonary system structure and function. Our data show that hMSCs may be a promising therapeutic strategy for the treatment of PAH in the future.

## Author Contributions

AA, PP-C, GM, MdS, LM, TM, AS, JV, PR-d-C, BG, VC, AF, PS, MM, and TF contributed to the acquisition and analysis of data. RM-O, MT, RS and GZ-S contributed to the design of the work and manuscript.

## Conflict of Interest Statement

The authors declare that the research was conducted in the absence of any commercial or financial relationships that could be construed as a potential conflict of interest. The reviewer BS and handling Editor declared their shared affiliation.
