# Supplementary figures and images for: Human Mesenchymal Stem Cell Therapy Reverses Su5416/Hypoxia-Induced Pulmonary Arterial Hypertension in Mice
Source: Front Pharmacol. 2018 Dec 6;9:1395. doi: 10.3389/fphar.2018.01395 (PMC6291748; doi:10.3389/fphar.2018.01395)

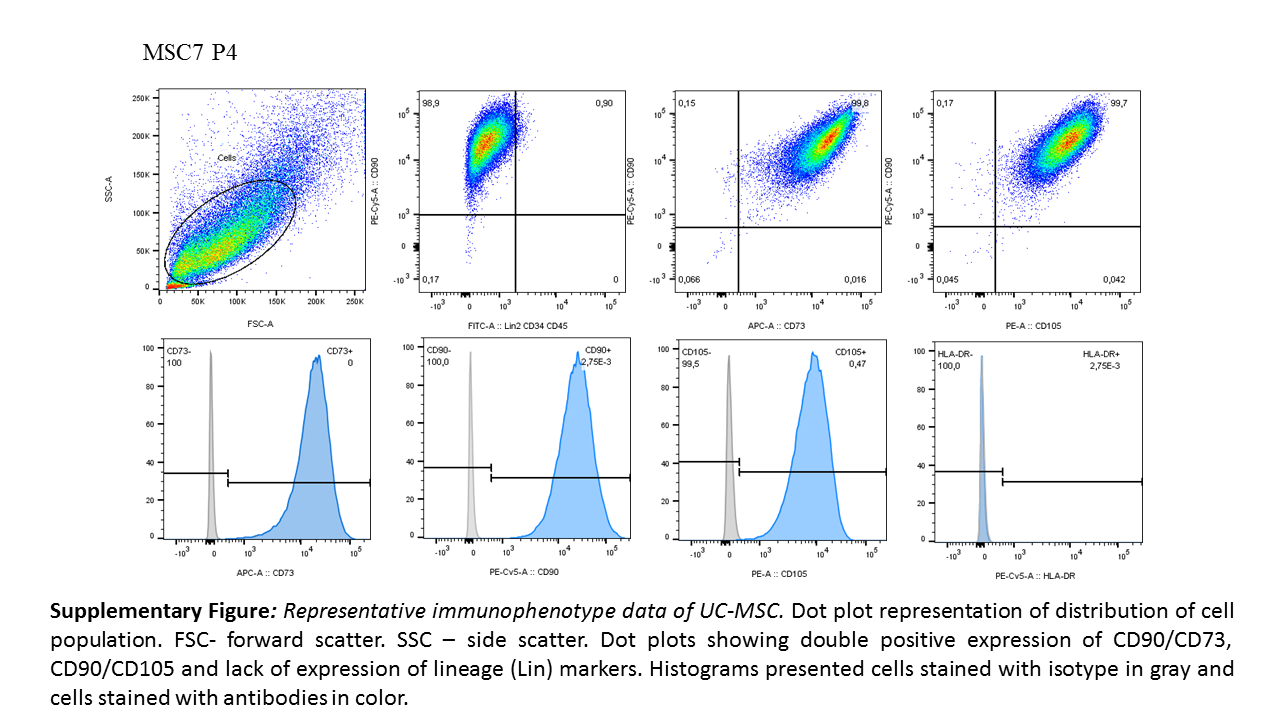

Supplement: Supplementary file 1 [file Image_1.TIF]

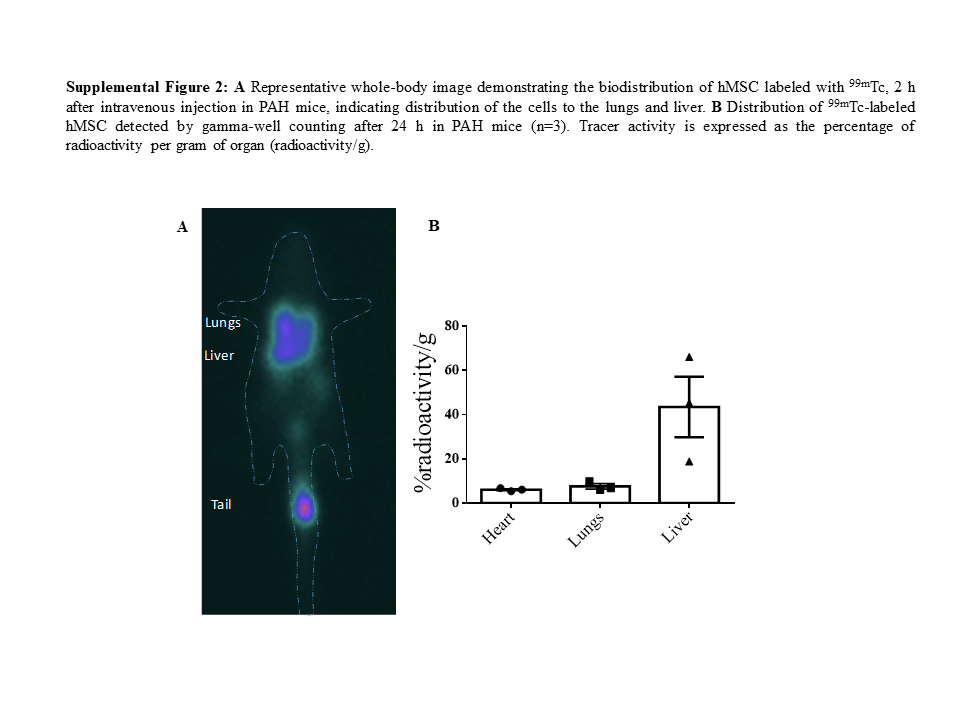

Supplement: Supplementary file 2 [file Image_2.TIF]

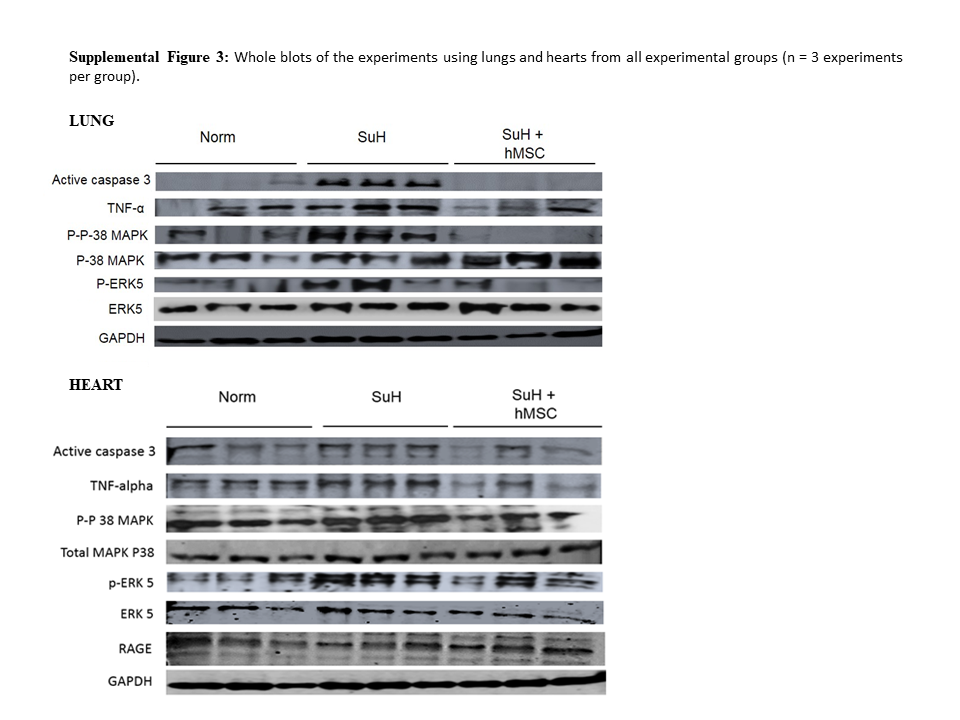

Supplement: Supplementary file 3 [file Image_3.TIF]
